# Co-Immobilization of Lipases with Different Specificities for Efficient and Recyclable Biodiesel Production from Waste Oils: Optimization Using Response Surface Methodology

**DOI:** 10.3390/ijms24054726

**Published:** 2023-03-01

**Authors:** Qian Wang, Rongjing Zhang, Maogen Liu, Lin Ma, Weiwei Zhang

**Affiliations:** State Key Laboratory of High-Efficiency Utilization of Coal and Green Chemical Engineering, School of Chemistry and Chemical Engineering, Ningxia University, Yinchuan 750021, China

**Keywords:** biodiesel, lipase, co-immobilization, process optimization, waste oils

## Abstract

Lipase-catalyzed transesterification is a promising and sustainable approach to producing biodiesel. To achieve highly efficient conversion of heterogeneous oils, combining the specificities and advantages of different lipases is an attractive strategy. To this end, highly active *Thermomyces lanuginosus* lipase (1,3-specific) and stable *Burkholderia cepacia* lipase (non-specific) were covalently co-immobilized on 3-glycidyloxypropyltrimethoxysilane (3-GPTMS) modified Fe_3_O_4_ magnetic nanoparticles (co-BCL-TLL@Fe_3_O_4_). The co-immobilization process was optimized using response surface methodology (RSM). The obtained co-BCL-TLL@Fe_3_O_4_ exhibited a significant improvement in activity and reaction rate compared with mono and combined-use lipases, achieving 92.9% yield after 6 h under optimal conditions, while individually immobilized TLL, immobilized BCL and their combinations exhibited yields of 63.3%, 74.2% and 70.6%, respectively. Notably, co-BCL-TLL@Fe_3_O_4_ achieved 90–98% biodiesel yields after 12 h using six different feedstocks, demonstrating the perfect synergistic effect of BCL and TLL remarkably motivated in co-immobilization. Furthermore, co-BCL-TLL@Fe_3_O_4_ could maintain 77% of initial activity after nine cycles by removing methanol and glycerol from catalyst surface, accomplished by washing with *t*-butanol. The high catalytic efficiency, wide substrate adaptability and favorable reusability of co-BCL-TLL@Fe_3_O_4_ suggest that it will be an economical and effective biocatalyst for further applications.

## 1. Introduction

With the rapid development of global modernization and industrialization, the worldwide consumption of energy is progressively increasing every year, consequently inducing ecological damage and serious environmental impacts [1,2]. Currently, fossil fuels provide approximately 70–80% of the total energy demand, leading to a serious carbon emissions crisis and depletion of non-renewable resources [3]. Therefore, the development of alternative renewable resources is imperative to ensure continuous energy supply and sustainable development. Biodiesel is considered to be the most suitable candidate for replacing petroleum-derived fuel because it is eco-friendly, biodegradable and technically feasible [4,5]. Additionally, biodiesel has superior combustion qualities with low sulfur and aromatic concentrations, while reducing the emission of greenhouse gases and particulates [6]. Furthermore, because of possessing similar characteristics to those of petroleum diesel, biodiesel can be used in the current compression ignition engines with minor modification [6].

The global output of biodiesel is steadily increasing, with about 41.4 billion liters expected in 2025 [7]. Among various procedures for biodiesel production, transesterification of oils and short-chain alcohols is the most efficient and economic approach using a wide variety of natural feedstocks including vegetable oils, animal fats, waste oils, and oleaginous microorganisms [8]. Although the majority of global biodiesel is derived from enabled oils at present, the use of cheap waste oils is economically valuable in light of production cost and food security [9]. However, due to the poor quality, the conventional chemically catalyzed transesterification of waste oils has been restricted, including saponification and consequent low yields in alkaline-catalyzed reactions, as well as low reaction rate and corrosive nature in acid-catalyzed reactions [10]. Lipase-catalyzed transesterification is an attractive and sustainable approach for biodiesel production, offering the advantages of compatible feedstock flexibility, environmentally friendly, energy-efficient, and easy separation of products [11,12]. Typically, immobilized lipases are required for economically viable applications to reduce the cost, owing to the improved operational stability and easy recovery [13,14]. In recent years, diverse immobilization strategies and carrier materials have been developed to obtain highly active immobilized lipases for biodiesel production [15,16]. Furthermore, well-designed immobilization protocols could open up integrated ways for increasing enzyme activity and stability, reducing inhibition, facilitating product isolation, and even controlling reaction process [17]. 

Lipases belong to the class of serine hydrolase with high specificity toward different substrates, especially for heterogeneous substrates such as oils [18,19]. During the modification of oils, the substrate mixtures are composed of different glycerides, including tri-, di-, or monoglycerides formed by diverse free fatty acids [20]. In the reaction of such complex components, lipases present different selectivities, including typoselectivity for fatty acids, regioselectivity for positional ester groups (such as 1,3-specific and 2-specific), and enantioselectivity [21]. Moreover, some of the glycerides formed in the reaction may have an inhibitory effect on the lipase, which is the ideal catalyst for the major components of the starting oil [22]. Therefore, the combined use of lipases with different specificities would have a significantly improvement in full modification of oils [22]. Lipase mixtures, in free or immobilized form, have been applied in the production of biodiesel to increase efficiency and yields [23]. Miotti et al. [24] reported the combination of lipases from *Burkholderia cepacia* (BCL) and *Thermomyces lanuginosus* (TLL) immobilized onto a silica hydroxyethylcellulose matrix in the transesterification of palm kernel oil. In this work, the lipases were packed separately in a continuous two-stage reactor, achieving highest volumetric productivity of 415.3 μmol gcat^−1^ h^−1^. The positive synergistic effect could be attributed to the complementary advantage of the 1,3-specific TLL and non-specific BCL. In another study, Binhayeeding et al. [25] demonstrated the enhanced biodiesel yield using a mixture of lipases from *Candida rugosa* (CRL) and *Rhizomucor miehei* (RML) immobilized on polyhydroxybutyrate, obtaining a maximum biodiesel yield of 96.5% within 24 h.

Co-immobilization is a promising proposal for accelerating the speed of reaction, taking the advantage of reduced lag time that occurs when different enzymes are used in physical combination [26,27]. According to Yao et al. [28], covalently co-immobilized CRL and TLL on polydopamine coated Fe_3_O_4_ nanoparticles exhibited superior catalytic activity in the preparation of functional oils compared to the combination of immobilized monolipases. Shahedi et al. [29] developed the co-immobilization of lipase from *Candida antarctica* B (CALB) and TLL on epoxy functionalized silica gel. The co-immobilized lipases could obtain a biodiesel yield of 94% through the transesterification of palm oil within 24 h. In another study, RML and CALB were co-immobilized on amine-functionalized silica-coated magnetic nanoparticles, which produced a biodiesel yield of up to 99% from waste cooking oil after 72 h [30]. Abdulmalek et al. [31] reported the covalent co-immobilization of *Rhizopus oryzae* lipase (ROL) and CRL on a four-arm polyethylene glycol amine polymer. The co-immobilized lipases were applied to prepare biodiesel from waste cooking oil under ultrasound conditions resulting in 97.64% biodiesel conversion rate within 120 min. However, it will be a great challenge to find the appropriate conditions for all the enzymes involved to have high activity and stability in co-immobilization. Meanwhile, co-immobilization of different enzyme may risk abandoning the entire biocatalyst when only one of the enzymes is inactivated [27].

Considering different enzymes bearing different stabilities, a reasonable optimization of the co-immobilization conditions is of critical importance to completely motivate the advantages of each enzyme. For this purpose, the response surface methodology (RSM) is a powerful tool for maximizing the performance of co-immobilized enzymes, combining mathematical and statistical techniques to determine the optimum operating conditions. Additionally, the mathematical model of RSM facilitates the analysis of interactions between variables and their effects on the response in a few experiments [32,33]. Therefore, it has been widely used in recent years for the development and optimization of biological and chemical processes [34,35]. However, there are limited reports of applying RSM to optimize the co-immobilization process of enzymes.

This study aims at developing efficient and reusable co-immobilized lipases for waste oil-derived biodiesel production. To this end, for the first time, highly active TLL (1,3-specific) and stable BCL (non-specific) were co-immobilized on 3-GPTMS-modified Fe_3_O_4_ MNPs. The functional magnetic carriers were selected to facilitate the recovery and improve the operational stability of biocatalyst. A 3-level–3-factor Box–Behnken Design (BBD) was applied to design the experiments and RSM was exploited to optimize co-immobilization process. The RSM approach has been commonly used to maximize biodiesel yield; however, its application in the optimization of immobilization conditions has rarely been reported. Various variables that influence the activity of co-immobilized lipases and their interactions are described in detail. Moreover, the synergistic advantage of co-immobilized TLL and BCL in biodiesel production from waste oil is investigated under different reaction conditions. Finally, the reusability of co-immobilized TLL and BCL in biodiesel production are performed. To the best of our knowledge, this is the first study to demonstrate that the synergistic effect between TLL and BCL is further stimulated in co-immobilization.

## 2. Results and Discussion

### 2.1. Preparation and Characterization of Co-Immobilized Lipases

**Figure 1:**
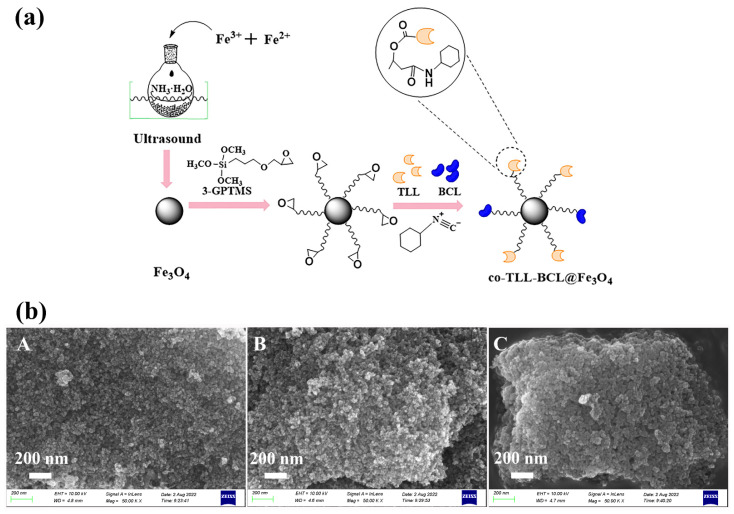
(**a**) Schematic preparation and (**b**) scanning electron microscopy images ((**A**) Fe_3_O_4_ MNPs; (**B**) 3-GPTMS-modified Fe_3_O_4_ MNPs; (**C**) co-BCL-TLL@Fe_3_O_4_) of magnetic carriers and co-immobilized lipases (co-BCL-TLL@Fe_3_O_4_).

As illustrated in Figure 1a, the preparation of co-BCL-TLL@Fe_3_O_4_ was carried out according to a three-step procedure. The efficient co-immobilization of TLL and BCL on 3-GPTMS modified Fe_3_O_4_ MNPs was achieved by a multicomponent reaction between epoxy group, cyclohexyl isocyanate and carboxy residues of lipase [29,36]. The high active nucleophilic attack of isocyanate group to epoxy group availably accelerated the binding of lipase. 

As particle shape, size and surface morphology could significantly affect the catalytic activity of immobilized enzyme, synthesized magnetic carriers and co-BCL-TLL@Fe_3_O_4_ were characterized by SEM observation (Figure 1b). Compared to magnetic carriers, co-BCL-TLL@Fe_3_O_4_ maintained a uniform spherical shape with slightly increase on average diameter, suggesting homogeneous core–shell covering of 3-GPTMS and lipases. Meanwhile, the microscale dimensions of co-BCL-TLL@Fe_3_O_4_ implied a more homogeneous biocatalyst in reaction mixture, facilitating an easy handing and recovery [37]. 

**Figure 2:**
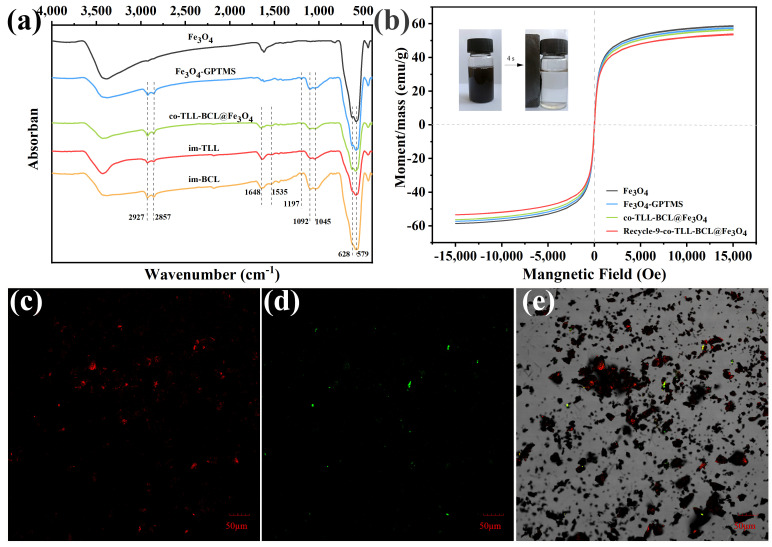
(**a**) Fourier transform infrared spectra, (**b**) magnetic hysteresis loops and confocal laser scanning microscopy micrographs (**c**) at 488 nm excitation and (**d**) at 559 nm excitation, and (**e**) overlay (dark field and bright field) of co-immobilized lipases (co-BCL-TLL@Fe_3_O_4_).

As shown in Figure 2a, the functionalization of Fe_3_O_4_ MNPs and co-immobilization of lipases were confirmed using FT-IR spectra. The typical peaks of Fe_3_O_4_ at 579 cm^−1^ and 628 cm^−1^ in all samples are attributed to the stretching vibration of Fe-O bond. Compared to the spectrum of Fe_3_O_4_ MNPs, two strong broad bands presented at 1045 cm^−1^ and 1092 cm^−1^ are instigated by Si-O vibration, while new peaks at 1197 cm^−1^ are assigned to Si-OCH_3_ stretching [38], indicating the functionalization of Fe_3_O_4_ MNPs with 3-GPTMS. The characteristic peaks of C=O stretching (amide I) and N-H bending (amide II) at 1648 cm^−1^ and 1535 cm^−1^ prove the success binding of TLL and BCL on magnetic carrier. 

The magnetic features of magnetic carriers and co-BCL-TLL@Fe_3_O_4_ were explored by vibrating sample magnetometer (VSM) and illustrated in Figure 2b. Although the saturation magnetization (M_S_) values decreased slightly after core–shell coating of 3-GPTMS and lipases, the magnetic hysteresis loops of all magnetic nanocomposites exhibited characteristic zero coercivity and remanence, suggesting superparamagnetic behavior. Consequently, co-BCL-TLL@Fe_3_O_4_ displayed fast magnetic separation in reaction medium using an external magnet (inset of Figure 2b), while it could be easily dispersed again in the reaction after removal of the magnet and slightly shaking. Moreover, the M_S_ value of co-BCL-TLL@Fe_3_O_4_ decreased from 56.55 to 53.98 emu/g after nine runs of recycling, indicating the excellent recyclability and magnetic stability of co-BCL-TLL@Fe_3_O_4_ with long-time reuse. 

The spatial distribution and assembly of TLL and BCL co-immobilized on magnetic carriers were observed using confocal laser scanning microscopy. For this purpose, BCL and TLL were labeled with iso-Rhodamine B (RhB) and thiocyanate (FITC), respectively, before the co-immobilization procedure. As shown in Figure 2c,d, the red and green fluorescence derived from RhB and FITC could confirm the success co-immobilization and relatively uniformly distribution on magnetic carrier (Figure 2e).

### 2.2. Optimization of Co-Immobilization Conditions

In the present work, to obtain the best co-immobilized BCL and TLL (co-BCL-TLL@Fe_3_O_4_) in transesterification of WCO, effects of different co-immobilization conditions on the catalytic activity of co-BCL-TLL@Fe_3_O_4_ were evaluated by biodiesel yields in corresponding reactions. Meanwhile, optimization of significant parameters involved in co-immobilization was achieved using response surface methodology (RSM) with Box–Behnken design (BBD). The total amount of lipase bound to the magnetic carriers was calculated after co-immobilization depending on Bradford method, resulting in 100% immobilization yields under all conditions. Therefore, the corresponding biodiesel yield was set as the dependent variable in optimization of the co-immobilization process. The main factors affecting the catalytic activity of co-BCL-TLL@Fe_3_O_4_ in biodiesel production were selected as: amount of carrier (A), cyclohexyl isocyanate dosage (B), and BCL:TLL ratio (C). The biodiesel yields for 17 runs of experimental design and homologous predicted values are presented in Table 1, ranging from 56.95% to 92.60%. The experimental values in each group matched the relative predicted values in software, demonstrating the applicability of the proposed RSM model. To reveal the behavior of biodiesel yield as a function of three factors in co-immobilization, the quadratic polynomial regression equation calculated by BBD was presented in Equation (1). Synergetic (positive value) and antagonistic (negative value) effects of each factor were considered, including linear, two-factor interaction, and quadratic coefficients.

(1) $$Y=+89.68+7.57A+1.71B+6.35C+4.16AB+0.090AC-0.33BC-2.94A^{2}-12.73B^{2}-9.83C^{2}$$

**Figure 3:**
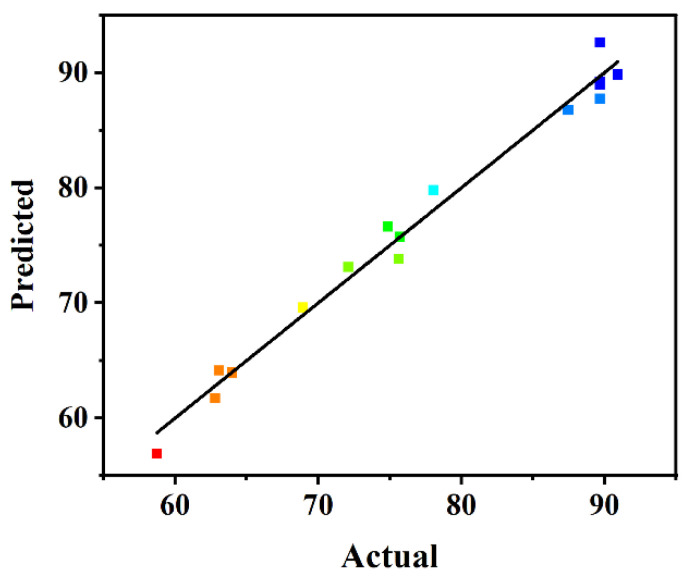
Plot of predicted versus actual biodiesel yield.

To confirm the statistical adequacy of the generated regression model, analyses of variance (ANOVA) were applied, and the results are summarized in Table 2. Considering the *p*-values, the individual variables with highly significant influence on biodiesel yield responses were the amount of carrier (A) and the BCL:TLL ratio (C), among which the amount of carrier (A) was the most sensitive factor affecting the biodiesel yield, possessing the highest F-value of 103.25. The quadratic polynomial model developed was statistically significant, with a *p*-value < 0.0001 and an F-value of 52.43. The precision of the model was further measured by adjusted determination coefficient. The R^2^ value of 0.9854 and adjusted R^2^ value of 0.9666 demonstrated the accuracy and reliability of the regression model. Additionally, the statistically non-significant lack of fit (*p* value of 0.2827) indicated the high fitness of the model. As shown in the plot distribution of predicted versus actual values (Figure 3), the biodiesel yield calculated from the model satisfactorily fitted to the experimental data, demonstrating the accuracy and reliability of the model.

**Figure 4:**
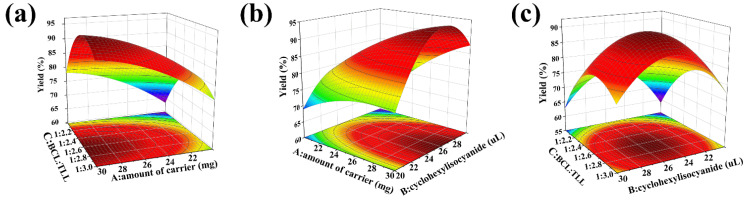
3D surface and 2D contour plots of interaction effects between two variables in co-immobilization: (**a**) amount of carrier vs. the BCL:TLL ratio; (**b**) amount of carrier vs. cyclohexyl isocyanate dosage; and (**c**) cyclohexyl isocyanate dosage vs. BCL:TLL ratio.

As shown in Figure 4, the 3D surface and 2D contour plots were generated to exemplify the interactions between the variables. Figure 4a displayed the interaction between the amount of carrier (A) and the BCL:TLL ratio (C) at a cyclohexyl isocyanate dosage of 25 μL. Enhanced biodiesel yield was observed when the amount of carrier increased from 20 to 30 mg and BCL:TLL ratio decreased from 1:2.00 to 1:2.68. However, the biodiesel yield decreased as the proportion of TLL increased further, as confirmed in the elliptical contour plot. As shown in Figure 4b, a similar behavior was observed in the interaction between amount of carrier (A) and cyclohexyl isocyanate dosage (B). The biodiesel yield increased when the amount of carrier increased from 20 to 30 mg and the cyclohexyl isocyanate dosage increased from 20.0 to 26.1 μL. Any further increase in cyclohexyl isocyanate dosage resulted in a decline in biodiesel yield. Coming to the cumulative effect of cyclohexyl isocyanate dosage (B) and BCL:TLL ratio (C), the biodiesel yield increased when the cyclohexyl isocyanate dosage and the BCL:TLL ratio rose to levels close to the middle level, and decreased with further increase in both factors (Figure 4c).

The optimal co-immobilization conditions predicted by the generated regression model were 30 mg of carrier, 26 μL cyclohexyl isocyanate dosage, and 1:2.6 ratio of BCL:TLL. The maximum biodiesel yield predicted by the BBD method was 96.02% after 12 h and the optimization of co-immobilization variables was further validated by experimental measurements performed in triplicate. The biodiesel yield observed from the experimental results was 92.02 ± 1.81%, which is in close agreement with the predicted value, indicating that the optimum conditions obtained were adequate for further application. 

**Figure 5:**
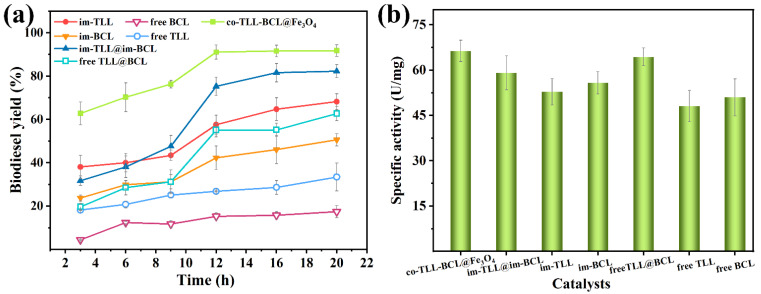
Catalytic activity of mono, combined-use and co-immobilized lipases during (**a**) transesterification of waste oil in n-hexane at methanol-to-oil molar ratio of 4:1 and 40 °C; (**b**) hydrolysis of p-nitrophenyl palmitate (p-NPP) in 0.1 M sodium phosphate buffer (pH 7.0) at 25 °C.

As shown in Figure 5a, the transesterifications of waste oil catalyzed by different lipase preparations were investigated in hexane as a function of time, including free lipases, individually immobilized lipases, combined use of free lipases (free BCL@TLL), combined use of individually immobilized lipases (im-BCL@im-TLL), and co-immobilized BCL and TLL (co-BCL-TLL@Fe_3_O_4_). Generally, co-BCL-TLL@Fe_3_O_4_ exhibited superior catalytic activity and rate to that of other counterparts, achieving a biodiesel yield of 91.1% after 12 h. After immobilization, the activity of free BCL and free TLL increased significantly, while TLL displayed higher catalytic activity and reaction rate compared to BCL. Therefore, the initial transesterification rates of free BCL@TLL and im-BCL@im-TLL showed a small decrease in relation to free TLL and im-TLL, which was due to the addition of less-efficient BCL. However, the advantages of both combinations including free BCL@TLL and im-BCL@im-TLL, came to the fore after 9 h, reaching biodiesel yields of 62.7% and 82.3% after 20 h, respectively. These results suggested the beneficial synergistic effect of BCL and TLL. More importantly, a significant improvement in activity and reaction rate was observed in the presence of co-BCL-TLL@Fe_3_O_4_ with a biodiesel yield of 62.8% after 3 h, indicating a prominent synergistic effect. 

Meanwhile, the specific activities of different lipase preparations were measured by the hydrolysis of p-nitrophenyl palmitate (p-NPP) and the results are presented in Figure 5b. The combination of different lipases, including free BCL@TLL and im-BCL@im-TLL, is superior to the use of individual lipases in terms of hydrolytic activity, implying the positive synergistic effect of BCL and TLL. Generally, co-BCL-TLL@Fe_3_O_4_ displayed the highest specific activity in hydrolyzing p-NPP among all tested counterparts. These results reveal that the co-immobilization of lipases acting in a synergistic way shows an advantage in terms of activity, owing to the reduction in lag time [26].

### 2.3. Optimization of Biodiesel Production

Next, the co-BCL-TLL@Fe_3_O_4_-catalyzed transesterification of waste oil and methanol was optimized under different conditions including solvents, molar ratio of methanol to oil, and temperatures. 

**Figure 6:**
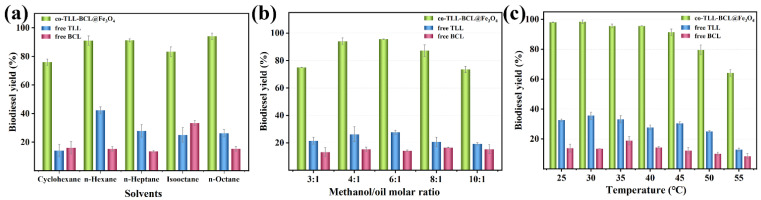
Effect of different transesterification conditions on biodiesel yield from waste oils: (**a**) solvent; (**b**) molar ratio of methanol to oil; (**c**) temperature.

Using proper solvents in biodiesel production could promote mass transfer efficiency while improving lipase activity and avoiding inhibition of short-chain alcohols [39]. Since lipases usually maintain good activity in hydrophobic solvents, several common hydrocarbon solvents were employed in the transesterification. As shown in Figure 6a, co-BCL-TLL@Fe_3_O_4_ could achieve more than 75% biodiesel yields after 12 h in all the solvents tested. A maximum yield of 94.1% was observed using n-octane as solvents, which might improve the miscibility of waste oil and methanol and decrease the deleterious effect of methanol [40]. Therefore, n-octane was chosen as the ideal reaction medium for further investigation.

As the acyl acceptor of transesterification, an appropriate increase in methanol concentration could raise the probability of collision between substrate and enzyme and enhance the reaction rate. Meanwhile, as a competitive inhibitor, excess methanol might induce aggregation and inactivation of enzyme [41,42], decreasing the reaction yield. Therefore, the molar ratio of methanol to oil (M:O) is a crucial parameter for enzymatic biodiesel production. According to Figure 6b, free TLL exhibited maximum yield at M:O of 6:1, while free BCL remained relatively constant with methanol concentration, indicating that methanol was better tolerated than free TLL. For co-BCL-TLL@Fe_3_O_4_, the biodiesel yield reached a maximum value at M:O of 6:1, and remained at a level of 87.2% at M:O of 8:1, suggesting a good tolerance to methanol. The optimal M:O of 6:1 was adopted for further optimization. 

The effect of reaction temperature on the enzymatic biodiesel production was explored by varying the temperature from 25 to 55 °C. As illustrated in Figure 6c, the optimal temperatures for free TLL and free BCL were 30 °C and 35 °C, respectively. For co-BCL-TLL@Fe_3_O_4_, 98.1% biodiesel yield was produced at 25 °C and the highest biodiesel yield of 98.4% was obtained at 30 °C after 12 h. When the reaction temperature increased from 30 °C to 45 °C, biodiesel yields declined slightly to 91.4%. At a temperature above 50 °C, the biodiesel yield decreased drastically due to the thermal inactivation of lipases. Hence, the optimum reaction temperature was set at 30 °C, implying an energy-saving biodiesel production. 

**Figure 7:**
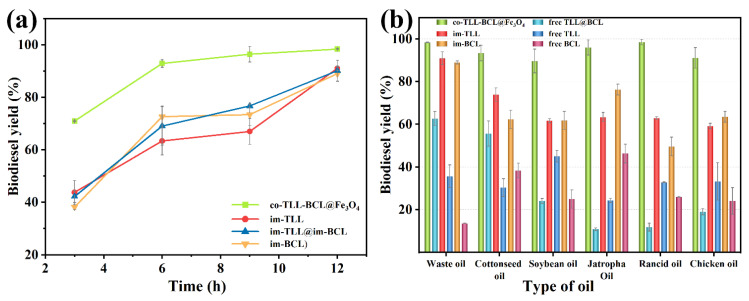
Comparison between mono, combined-use and co-immobilized lipases in biodiesel production under optimal conditions: (**a**) time-course of transesterification using waste oils in n-octane at methanol-to-oil molar ratio of 6:1 and 30 °C; (**b**) transesterification using different feedstocks.

In addition, a comparison between co-BCL-TLL@Fe_3_O_4_, individually immobilized lipases (im-TLL and im-BCL), and the combination of immobilized lipases in transesterification of waste oil was performed as a function of time in n-octane at 6:1 M:O and 30 °C. As shown in Figure 7a, co-BCL-TLL@Fe_3_O_4_ displayed significantly superior catalytic efficiency to im-TLL, im-BCL, and im-TLL@im-BCL, achieving 92.9% yield after 6 h and reaching the equilibrium stage after 9 h. Owing to the good tolerance of BCL to methanol, im-BCL exhibited comparable activity to im-TLL in n-octane at 6:1 M:O, compared to their performances in hexane at 4:1 M:O (Figure 5). 

Furthermore, co-BCL-TLL@Fe_3_O_4_ was applied to biodiesel production under optimal reaction conditions using various feedstocks, including edible vegetable oil (soybean oil), non-edible vegetable oil (cottonseed oil and jatropha oil), waste oil (waste cooking oil and rancid oil), and animal oil (chicken oil). The catalytic performances of different lipase counterparts for various oils were shown in Figure 7b. Compared to individually immobilized lipases and combined-use of lipases, co-BCL-TLL@Fe_3_O_4_ exhibited outstanding catalytic activity toward all tested oils with biodiesel yields exceeding 90% after 12 h. Conversely, the catalytic performances of free TLL and free BCL were obviously dependent on the type of oil, suggesting that lipases possessing different regioselectivity and typoselectivity in the transesterification with triacylglycerols. It can be inferred from these results that the co-immobilization of lipases differing in their selectivity could provide a fully functional and catalytic active biocatalyst for heterogeneous substrates.

**Figure 8:**
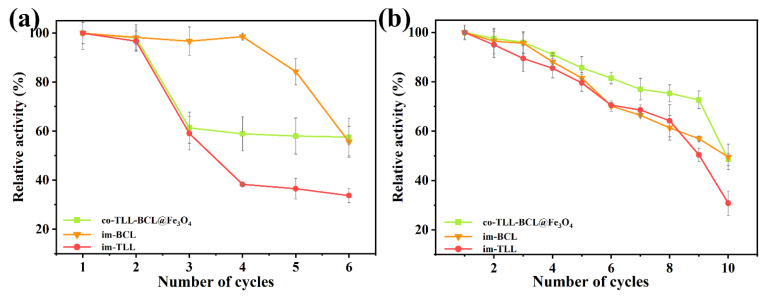
Reusability of individually immobilized lipases and co-immobilized lipases in the transesterification of waste oils into biodiesel at different conditions: (**a**) at methanol-to-oil molar ratio of 6:1; (**b**) at methanol-to-oil molar ratio of 4:1 (biocatalysts were washed thrice with *t*-butanol before reuse).

The magnetic carrier confers the convenience of co-immobilized lipases for recycling operation by rapid magnetic separation. The reusability of co-BCL-TLL@Fe_3_O_4_ and individually immobilized lipases in continuous transesterification cycles of waste oil was evaluated under optimal reaction conditions. After each cycle, immobilized lipases were washed three times with n-octane and reused in a fresh reaction mixture. As presented in Figure 8a, the activity of co-BCL-TLL@Fe_3_O_4_ and im-TLL decreased significantly after two repeated applications, whereas im-BCL maintained its initial activity in the fourth cycle, indicating that TLL is more prone to denaturation compared to BCL. The inactivation of lipases in biodiesel production might be due to the excess of methanol and the accumulation of glycerol [43]. However, as a kinetically controlled synthesis, the yield of transesterification is influenced by the properties of lipase and the concentration of nucleophile (methanol) [44]. Therefore, it is of great importance to balance the yield of biodiesel with the detrimental effect of methanol. To reduce the impact of methanol, the transesterification were carried out at 4:1 M:O. Meanwhile, to fully eliminate excess methanol and the generated glycerol, the immobilized lipases were wash with *t*-butanol prior to the n-octane wash in recycling. As shown in Figure 8b, co-BCL-TLL@Fe_3_O_4_ could maintain 77% of initial activity after nine cycles, suggesting a superior reusability, which might be attributed to the effective removal of methanol and glycerol by washing with *t*-butanol. This simple washing procedure offered a facile way to prevent the rapid deactivation of immobilized lipases, thereby extending the operational lifetime. 

Integrating lipases with different specificities by co-immobilization, the obtained co-BCL-TLL@Fe_3_O_4_ displayed better catalytic activity and reusability in biodiesel production compared to previously reported combined-use or co-immobilized lipases (Table 3), while exhibiting a wide adaptability to different oils. Moreover, the removal of excess methanol and generated glycerol from catalyst surface reduced the corresponding inactivation of lipase, resulting in an improved reusability. To eliminate the glycerol-induced inhibition of immobilized lipases, hydrophobic functionalization of carriers and ultrasound process are promising solutions to avoid accumulation of glycerol at source [45], and will be investigated in the follow-up research. Additionally, screening synergistic effects of different lipases and developing new strategies for co-immobilization of multi-lipases will be explored to improve the efficiency of biodiesel production.

## 3. Materials and Methods

### 3.1. Materials

Lipase from *Burkholderia cepacia* (BCL), *Thermomyces lanuginosa* (TLL), and fatty acid methyl ester standards were obtained from Sigma-Aldrich. Cyclohexyl isocyanate and 3-glycidyloxypropyltrimethoxysilane (3-GPTMS) were purchased from Aladdin Biochemical Technology Co., Ltd. (Shanghai, China). Waste oil (WO) was collected from a local restaurant with fatty acids composition of 5.69% oleic acid, 75.21% linoleic acid, 10.13% palmitic acid, and 8.97% stearic acid. The saponification value and acid value of WCO were determined to be 195.96 mg KOH/g and 7.75 mg KOH/g, respectively. All other reagents and solvents were of analytical or HPLC grade and obtained commercially.

### 3.2. Synthesis and Functionalization of Fe_3_O_4_ Magnetic Nanoparticles

Fe_3_O_4_ magnetic nanoparticles (MNPs) were synthesized by an ultrasonic-assisted reverse co-precipitation method following a previously described method [46] with a slight modification. Briefly, 2.70 g FeCl_3_·6H_2_O and 1.99 g FeCl_2_·4H_2_O were dissolved in 30 mL of distilled water, and added dropwise into 40 mL of ammonia solution (10%) under ultrasound irradiation at 60 °C for 30 min. Afterward, the obtained black Fe_3_O_4_ MNPs was washed with distilled water to neutral and lyophilized.

To functionalize Fe_3_O_4_ MNPs with 3-GPTMS, 1.0 g Fe_3_O_4_ MNPs was dispersed into 30 mL of dry toluene containing 1 mL of 3-GPTMS and 0.15 mL of triethylamine. The suspension was refluxed under argon atmosphere and stirred for 4 h. Then, the precipitate was obtained by magnetic separation, washed thoroughly with ethanol and distilled water, and lyophilized.

### 3.3. Preparation of Magnetic Co-Immobilized Lipases

The three-component reaction was used to co-immobilize BCL and TLL on 3-GPTMS functionalized Fe_3_O_4_ MNPs according to the method described by Mohammadi et al. [47]. BCL and TLL with different mass ratio were dissolved in 6.5 mL phosphate buffer (0.1 M, pH 7.0), following by the dispersion of 3-GPTMS functionalized Fe_3_O_4_ MNPs (25 mg). After incubation at 30 °C for 30 min, the co-immobilization was started by adding appropriate amount of cyclohexyl isocyanate. The mixture was shaken for 3 h, and then the co-immobilized BCL and TLL was collected by an external magnet, washed with distilled water, and freeze-dried. The total concentration of lipases was kept at 3 mg/mL. The immobilization yields of lipases was calculated by measuring the initial and residual protein concentration in supernatant using Bradford assay.

### 3.4. Transesterification Activity of Magnetic Co-Immobilized Lipases

The transesterification activity of magnetic co-immobilized lipases and free lipases were measured by enzymatic biodiesel production from waste cooking oil (WCO). Typically, the transesterification of WCO (0.25 g) and methanol were performed in 10 mL capped flask at methanol:oil molar ratio of 4:1. Methanol was added all at once, and the mixture was incubated with different lipase preparations under constant shaking at 250 rpm at 30 °C for 12 h. At the end of each batch, co-immobilized lipases were separated by an external magnet and aliquots of supernatant were analyzed by gas chromatography. In a multiple recycling procedure, co-immobilized lipases were washed thrice with *t*-butanol and solvents, respectively, and subsequently redispersed in the fresh reaction mixture. 

The samples from the transesterification of waste oil were analyzed by gas chromatography (Fuli 9790 plus, China) equipped with flame ionization detector (FID) and a KB-FFAP capillary column (60 m × 0.32 mm × 0.25 μm). Methyl tridecanoate was served as an internal standard to quantify the amount of FAMEs. The oven program was set as follows: held at initial value of 160 °C for 2 min, then heated to 240 °C at 10 °C/min and held at 240 °C for 8 min. The biodiesel yield (%) was calculated using Equation (2).

(2) $$Biodiesel\;yield=\frac{\sum mass\;of\;FAMEs\;determined\;by\;GC}{mass\;of\;oil}\times 100\%$$

### 3.5. Hydrolysis Activity of Magnetic Co-Immobilized Lipases

The specific activity of magnetic co-immobilized lipases and free lipases were measured by the hydrolysis of p-nitrophenyl palmitate (p-NPP) in 0.1 M sodium phosphate buffer (pH 7.0) at 25 °C. The substrate solution consisted of 8 mM p-NPP and 0.4% Triton X-100 in 0.1 M phosphate buffer. Then, 100 μL of the lipase suspension was added to 2.4 mL of substrate solution under magnetic stirring for 2 min. An UV-1800 ultraviolet-visible spectrophotometer (Shimadzu, Kyoto, Japan) was used in the concentration quantification of released p-nitrophenol (p-NP) at 410 nm. Specific activity (U/mg) was assessed as 1 μmol of p-NPP hydrolyzed per min per mg of lipase under the assay conditions.

### 3.6. Optimization of Co-Immobilization Conditions Using Response Surface Methodology

Optimization of the co-immobilization conditions was investigated using the 3-level 3-factorial Box–Behnken design (BBD) of Response Surface Methodology (RSM) in the Design Expert 10 software package [48]. The three identified independent variables were A: mass of carrier (20–30 mg); B: cyclohexyl isocyanate dosage (20–30 μL); and C: BCL:TLL ratio (1:2–1:3); and the response value was the biodiesel yield. The independent variables were coded to three levels: Low (−1), Medium (0), and High (1). The designed levels of each independent variable were derived from previous single-factor investigations and are shown in Table 4. To reduce errors caused by systematic trends in the variables, a total of 17 experimental runs were performed at random and repeated 3 times. To investigate the effect of each independent variable on the response and their interactions, a mathematical expression of the obtained responses was revealed by a second-order polynomial equation described as shown in Equation (3), below.

(3) $$Y=\beta_{0}+\sum_{i=1}^{3}\beta_{i}X_{i}+\sum_{i=1}^{3}\beta_{ii}X_{i}^{2}+\sum_{i=1}^{2}\sum_{j=i+1}^{3}\beta_{ij}X_{i}X_{ij}$$

where Y is the biodiesel yield; *X_i_*, *X*_1_, *X*_2_, … *X_j_* are the independent variables; *β*_0_ is constant; *β_i_*, *β_ii_* and *β_ij_* are coefficients of linear, quadratic and interactions, respectively. To confirm the optimum conditions for co-immobilization, analysis of variance (ANOVA), regression analysis, response surface and contour plots were used. The significance of the mathematical model was tested using *p*-values and Lack-of-Fit values. 

### 3.7. Characterization of Co-Immobilized Lipases

The Fourier-transform infrared (FTIR) spectra of co-immobilized lipases were acquired in the range of 4000–400 cm^−1^ using a Perkin Elmer Spectrum Two spectrometer. The size and surface morphology of co-immobilized lipases were recorded with a scanning electron microscope (MIRA4, TESCAN, Brno, Czech Republic). The magnetic properties of co-immobilized lipases were analyzed using a vibrating sample magnetometer (MicroSense EZ9, Lowell, MA, USA). A 5 mL volume of lipase solution (BCL or TLL, 2 mg/mL) was mixed with 100 μL of iso-Rhodamine B (RhB) or thiocyanate (FITC) solution (dimethyl sulfoxide, 1 mg/mL), respectively. After incubation in dark at 30 °C for 24 h, the unreacted FITC and RhB were removed by dialysis with distilled water for 48 h. Then, RhB-labeled BCL and FITC-labeled TLL were co-immobilized and observed using a confocal laser scanning microscope (CLSM, Olympus FV1200, Tokyo, Japan). 

## 4. Conclusions

In this study, for the first time, 1,3-specific TLL and non-specific BCL were covalently co-immobilized on 3-GPTMS modified Fe_3_O_4_ MNPs. The co-immobilization process was optimized using RSM with a 3-level–3-factor BBD. Compared with mono lipase and combined-use lipases, co-BCL-TLL@Fe_3_O_4_ exhibited a significant improvement in activity and reaction rate, indicating the beneficial synergistic effect of BCL and TLL remarkably motivated in co-immobilization. Various factors influencing co-BCL-TLL@Fe_3_O_4_-catalyzed biodiesel production were investigated, resulting in a maximum yield of 98.4% within 12 h under optimal conditions. Significantly, co-BCL-TLL@Fe_3_O_4_ displayed superior activity at 30 °C, suggesting a low-energy-consuming enzymatic process. Furthermore, co-BCL-TLL@Fe_3_O_4_ was applied to biodiesel production using six different feedstocks, with biodiesel yields exceeding 90% were obtained after 12 h in all tested oils. The wide substrate adaptability demonstrated the perfect complementation of BCL and TLL in terms of specificity. Owing to the effective removal of methanol and glycerol by washing with *t*-butanol, co-BCL-TLL@Fe_3_O_4_ could maintain 77% of initial activity after nine cycles, suggesting superior reusability. Therefore, this study provides a highly efficient, magnetically separable and reusable biocatalyst for sustainable production of biodiesel from abundant oils.

## Figures and Tables

**Table 1:** Experimental design variables with actual and predicted biodiesel yield (%).

Run	Actual Value of Variables			Actual Biodiesel Yield (%)	Predicted Biodiesel Yield (%)
	A: Amount of Carrier (mg)	B: Cyclohexyl Isonitrile Dosage (μL)	C: BCL:TLL Ratio		
1	25	25	1:2.5	92.60	89.68
2	25	20	1:3.0	73.14	72.09
3	20	25	1:3.0	73.85	75.60
4	25	25	1:2.5	87.74	89.68
5	25	20	1:2.0	56.95	58.73
6	30	30	1:2.5	86.75	87.45
7	20	20	1:2.5	69.60	68.90
8	20	30	1:2.5	63.95	63.98
9	25	30	1:2.0	61.75	62.80
10	30	20	1:2.5	75.75	75.72
11	25	25	1:2.5	89.92	89.68
12	30	25	1:2.0	79.79	78.04
13	25	25	1:2.5	88.92	89.68
14	25	30	1:3.0	76.63	74.85
15	20	25	1:2.0	64.15	63.07
16	30	25	1:3.0	89.85	90.93
17	25	25	1:2.5	89.22	89.68

**Table 2:** Regression analysis of variance (ANOVA) for the quadratic polynomial model.

Source	Sum of Squares	df	Mean Square	F-Value	*p*-Value Prob > F	
Model	2097.32	9	233.04	52.43	<0.0001	significant
A-Amount of carrier	458.89	1	458.89	103.25	<0.0001	
B-Cyclohexyl isonitrile dosage	23.26	1	23.26	5.23	0.0560	
C-BCL:TLL ratio	322.96	1	322.96	72.67	<0.0001	
AB	69.31	1	69.31	15.59	0.0055	
AC	0.032	1	0.032	7.29 × 10^−3^	0.9343	
BC	0.43	1	0.43	0.097	0.7651	
A2	36.33	1	36.33	8.17	0.0244	
B2	682.33	1	682.33	153.52	<0.0001	
C2	407.07	1	407.07	91.59	<0.0001	
Residual	31.11	7	4.44			
Lack of Fit	17.97	3	5.99	1.82	0.2827	not significant
Pure Error	13.14	4	3.28			
Cor Total	2128.43	16				
R2	0.9854					
Adjusted R2	0.9666					
Predicted R2	0.8552					

**Table 3:** Comparison of catalytic performances of combined-use and co-immobilized lipases in biodiesel production.

Lipase	Support	Method	Oil	Reaction Conditions	Yield	Reusability	Ref.
*Candida rugosa* (CRL) and *Rhizomucor miehei* (RML)	poly hydroxybutyrate (combined use)	Adsorption	Waste cooking oil	1% of mixed lipase, 5% water content, methanol/oil = 6:1, 45 °C, 24 h	96.5%	6 cycles, 60% relative activity	[9]
*Candida rugosa* (CRL) and *Rhizomucor miehei* (RML)	poly hydroxybutyrate (combined use)	Adsorption	Chicken waste oil	2.5% wt enzyme, 5% water content, methanol/oil = 6:1, 40 °C, 12 h	97.1%	7 cycles, more than 50% relative activity	[11]
*Burkholderia cepacia* (BCL) and *Thermomyces lanuginosus* (TLL)	silica hydroxyethylcellulose matrix (combined use)	Covalent bonding	Palm kernel oil	25% wt enzyme, ethanol/oil = 8:1, 45 °C, space-time of 16 h	98%	-	[13]
*Rhizomucor miehei* (RML) and *Candida antarctica* B (CALB)	epoxy-functionalized silica gel (co-immobilization)	Covalent bonding	Palm oil	-, water 3% (*w*/*w* of oil) methanol/oil = 5.9 (2 steps), 40 °C, 33.5 h CALB:RML ratio (2.5:1),	88%	-	[8]
*Candida antarctica* B (CALB) and *Thermomyces lanuginose* (TLL)	epoxy-functionalized silica gel (co-immobilization)	Covalent bonding	Palm oil	-, methanol/oil = 2.3, t-butanol (45 wt%), 47 °C, 24 h	94%	-	[10]
*Burkholderia cepacia* (BCL) and *Thermomyces lanuginosus* (TLL)	epoxy-functionalized magnetic particles (co-immobilization)	Covalent bonding	Waste oil	lipase 8% (*w*/*w* of oil), no water, methanol/oil = 6:1, 30 °C, 12 h	98.5%	9 cycles, 77% relative activity	This work

-: no data available.

**Table 4:** The experimental matrix of Box–Behnken design.

Factors (Independent Variable)	Levels		
	Low (−1)	Medium (0)	High (1)
Amount of carrier (mg)	20	25	30
Cyclohexyl isonitrile dosage (μL)	20	25	30
BCL:TLL mass ratio	1:2.0	1:2.5	1:3.0

## Data Availability

Not applicable.
